# Effect of Epidural Analgesia During Low-Risk Labor and Delivery on Cerebral Oximetry in Term Neonates Measured by Near-Infrared Spectroscopy: A Pilot Study

**DOI:** 10.3390/jcm15093404

**Published:** 2026-04-29

**Authors:** María Teresa Gómez-Riesco Tabernero de Paz, Ana Garzón-Sánchez, Carlos Ricardo Vargas-Chiarella, José Alfonso Sastre-Rincón, Regina Ruiz de Viñaspre-Hernandez, Noelia Navas-Echazarreta, José Carlos Garzón-Sánchez

**Affiliations:** 1 Department of Nursing and Physiotherapy, University of Salamanca, 37007 Salamanca, Spain; mtgomezt@usal.es; 2 Department of Preventive Medicine and Quality Management, Gregorio Marañón University Hospital, 28007 Madrid, Spain; ana.garzon@salud.madrid.org; 3 Department of Anesthesiology and Resuscitation, University Assistance Complex of Salamanca, 37007 Salamanca, Spain; cvargaschiarella@gmail.com (C.R.V.-C.); jasastre@saludcastillayleon.es (J.A.S.-R.); jcgarzon@usal.es (J.C.G.-S.); 4 Research Group in Care and Health (GRUPAC), Department of Biomedical Sciences and Health Specialties, University of La Rioja, 26006 Logroño, Spain; regina.ruiz-de-vinaspre@unirioja.es

**Keywords:** epidural analgesia, obstetrical analgesia, term infant, newborn, near-infrared spectroscopy

## Abstract

**Background**: Epidural obstetric analgesia is the standard of care for labor pain relief; however, its impact on neonatal cerebral oximetry remains debated. **Objective**: We aimed to evaluate whether epidural analgesia modifies cerebral regional oxygen saturation (CrSO_2_), measured by near-infrared spectroscopy (NIRS), in term neonates from low-risk deliveries. **Methods**: We conducted a prospective comparative observational cohort study, including 48 term newborns: 25 delivered under epidural analgesia (EA) and 23 without epidural analgesia (NE). CrSO_2_ was monitored using NIRS (INVOS 5100C, Somanetics/Medtronic, Troy, MI, USA; OxyAlert NIRSensor Cerebral/Somatic Infant–Neonatal Sensor CNN/SNN) during the neonatal transition up to 15 min after birth (primary outcome), and its relationship with neonatal well-being parameters (umbilical cord pH, Apgar score, and other analytical and obstetric indicators) was explored. **Results**: Median CrSO_2_ at 15 min was 79.52 [76.40–82.64] in the EA group and 78.65 [74.21–83.09] in the NE group. Both groups exhibited a similar temporal pattern characterized by a progressive increase, a peak at 10 min, and stabilization by 15 min. Mean (SD) CrSO_2_ in EA/NE were: 2 min, 57.64 (14.8)/60.04 (14.4); 5 min, 79.56 (10.9)/79.39 (12.2); 10 min, 82.28 (8.1)/81.13 (9.7); 15 min, 79.52 (7.6)/78.65 (10.3). No significant between-group differences were detected at any time point using a linear mixed model (*p*-values: 2 min, 0.57; 5 min, 0.96; 10 min, 0.66; 15 min, 0.74). **Conclusions**: These findings indicate that epidural obstetric analgesia does not alter cerebral oximetry parameters in term neonates from low-risk deliveries during the early transitional period.

## 1. Introduction

Neuraxial epidural analgesia has demonstrated its safety as the standard method for labor pain control and is recommended by the World Health Organization. It is used by approximately 30% and 73% of women in labor in the United Kingdom and the United States, respectively, with a steady increase in its use worldwide [1,2,3]. Nevertheless, detailed information on its effects on neonatal outcomes remains limited and controversial, and may vary according to the type of analgesia, drugs, doses used, and timing of administration.

Although effective pain relief may improve the maternal stress response during labour, neuraxial analgesia can influence the fetus through both direct and indirect mechanisms. Direct effects are related to placental transfer of local anaesthetics and opioids, which generally have minimal impact on fetal heart rate and rarely cause neonatal respiratory depression. Indirect effects, more frequently reported, are mainly associated with maternal physiological changes induced by neuraxial blockade, including hypotension, alterations in catecholamine levels, increased uterine tone, and reduced uteroplacental perfusion, which may lead to transient fetal heart rate abnormalities. Consequently, careful maternal and fetal monitoring is recommended following initiation and maintenance of epidural analgesia [4].

Most effects of obstetric analgesia on the newborn are fortunately transient, without long-term consequences, underscoring the importance of individualized analgesia based on maternal and neonatal needs, as well as appropriate neonatal monitoring to detect any adverse effects of analgesia in the neonate.

Cerebral oximetry monitoring using NIRS technology in the delivery room during the immediate fetal-to-neonatal transition period has been widely employed. Early detection of low cerebral oxygenation during this period through such monitoring may reduce the rate of adverse neonatal outcomes by enabling appropriate therapeutic interventions before overt clinical changes occur. However, knowledge of the parameters that influence cerebral oxygenation during the immediate transition in uncomplicated deliveries remains limited.

Studies on the effect of mode of delivery on neonatal cerebral oximetry have yielded mixed results, as have perinatal outcomes more broadly [5,6,7,8,9] with reports including an increased risk of neonatal resuscitation and admission to the neonatal intensive care unit [10]. No studies assessing the effect of epidural analgesia during labor and delivery on cerebral oximetry in term neonates have been found. Few studies have investigated the impact of maternal anaesthesia on neonatal cerebral oxygenation, mainly focusing on the indirect effects on neonatal wellbeing related to maternal haemodynamic alterations induced by general or spinal anaesthesia during cesarean delivery [11,12,13,14,15,16].

The potential effects of epidural analgesia on neonatal cerebral oximetry may be attributable to three main factors: a reduction in oxygen saturation in neonatal cerebral blood, particularly when high doses of anesthetics are used; changes in fetal heart rate; and reduced uterine blood flow due to hemodynamic alterations, with subsequent impact on neonatal cerebral oxygenation. However, such associations have important limitations, often due to heterogeneity in study design (study type, population selection, etc.) as well as differences at the level of the hospital care provider—factors that can influence the quality of anesthetic, obstetric, and neonatal care [17,18].

Therefore, the aim of the present study was to evaluate the potential correlation between epidural analgesia and cerebral regional oxygen saturation (CrSO_2_), cerebral fractional tissue oxygenation extraction (cFTOE), and cerebral tissue oxygen extraction (cTOE), measured with NIRS, as well as with Apgar scores, umbilical cord blood gas parameters (pH, partial pressure of O_2_ [pO_2_], partial pressure of CO_2_ [pCO_2_]), and other metabolic variables in term neonates from low-risk deliveries at 15 min after birth.

We hypothesize that obstetric epidural analgesia does not modify neonatal cerebral oxygenation, measured as CrSO_2_ by NIRS, during the first 15 min of transition to extrauterine life.

## 2. Materials and Methods

We conducted a prospective comparative observational cohort study assessing the condition of term newborns through cerebral oximetry monitoring up to 15 min after birth in low-risk deliveries with epidural analgesia (EA group) and without epidural analgesia (NE group). The measurement and analysis of the variables were carried out exclusively by the principal investigator, who was aware of the group assignments. This methodological decision was made to avoid inter-observer variability and to ensure uniformity in data recording, thereby preserving the stability and internal reproducibility of the data. Deliveries were carried out by a professional other than the principal investigator, in the labor ward of the Department of Obstetrics and Gynecology at the Complejo Asistencial Universitario de Salamanca (CAUSA), Spain, from September 2017 to December 2021. The study adhered to the principles of the Declaration of Helsinki, and written informed consent was obtained from participants prior to delivery and study inclusion to safeguard their integrity and well-being. The research protocol (ID: CEIC: PI 8706/2017) was reviewed and approved by the Research Ethics Committee for Medicinal Products of the Salamanca Health Area on 31 July 2017.

Eligible participants met the following inclusion criteria: low-risk pregnancy and delivery, gestational age between 37 and 42 weeks, and spontaneous onset of labor. Exclusion criteria comprised maternal medical history of important diseases, risk factors in the current pregnancy and intrapartum complications (Appendix A).

Participants were assigned by consecutive sampling to either the epidural analgesia group or the no epidural analgesia group. This approach was adopted because non-epidural deliveries were less frequent than epidural deliveries in routine practice. Randomization was not performed, being ethically and morally questionable in this context, since the decision to accept neuraxial analgesia for labor is made voluntarily by the pregnant woman after appropriate obstetric and anesthetic counseling. All eligible women were invited to participate, acknowledging that a smaller proportion would choose not to receive epidural analgesia. All available cases meeting the inclusion criteria during the study period were included; therefore, no a priori sample size calculation was undertaken, as we worked with the accessible population.

EA was administered to the women at the time of their request, coinciding with the onset of the active phase of labor, when cervical effacement was 100% and cervical dilation reached 3–4 cm.

The EA protocol followed the current guidelines at the CAUSA (University Assistance Complex of Salamanca). The catheter insertion technique was performed in compliance with relevant aseptic and safety measures. After confirming correct placement, the initial bolus was administered in fractions to allow for the detection of accidental intradural catheter placement, as well as any undesired motor block.

Maintenance epidural analgesia consisted of levobupivacaine 0.0625–0.125% combined with fentanyl 2 µg/mL, administered according to the continuous infusion, PCEA, or PIEB protocol.

The initial bolus consisted of 10 mL of levobupivacaine 0.125% plus fentanyl 5 µg/mL. Once adequate analgesia had been achieved and the absence of motor block or other complications had been confirmed, maintenance epidural analgesia was initiated with levobupivacaine 0.0625–0.125% combined with fentanyl 2 µg/mL, according to the therapeutic regimens detailed in Table 1. In the epidural analgesia group, the mean total fentanyl dose administered during labor was 93.99 µg (SD 46.64; 95% CI 74.73–113.24).

Periodic assessments of maternal status were conducted throughout the entire process, evaluating episodes of hypotension, the need for vasoactive drugs, the level of motor block using the Bromage scale, and the effectiveness of pain relief via the Visual Analogue Scale (VAS).

Women who did not receive epidural analgesia used non-pharmacological methods for pain relief such as free movement, fitball, breathing techniques and massage.

Neonatal outcomes included Apgar scores at 1, 5, and 10 min; umbilical cord arterial blood gas parameters (pH, partial pressure of oxygen, partial pressure of carbon dioxide, and arterial oxygen saturation [SaO_2_]); and cerebral oximetry parameters up to 15 min after birth. Umbilical cord arterial blood samples were collected 2 min after birth (following delayed cord clamping) and analyzed using the GEM Premier 4000 Analyzer (Instrumentation Laboratory Company – Bedford, Massachusetts, USA). Cerebral oximetry was measured using the INVOS 5100C monitor (Somanetics/Medtronic, Troy, MI, USA) with the OxyAlert NIRS Cerebral/Somatic Infant–Neonatal Sensor (CNN/SNN). The following parameters were recorded: cerebral regional tissue oxygen saturation (CrSO_2_), cerebral fractional tissue oxygen extraction (cFTOE), and cerebral tissue oxygen extraction (cTOE). Use of neonatal resuscitation measures and the need for transfer to the neonatal intensive care unit were also documented.

Continuous variables were summarized as mean ± standard deviation (SD) for normally distributed data and as median with interquartile range (IQR) for non-normally distributed data, after assessing normality with the Shapiro–Wilk or Kolmogorov–Smirnov tests. Categorical variables were presented as counts and percentages and compared using the χ^2^ test or Fisher’s exact test, as appropriate. Between-group comparisons were performed using Student’s *t* test or the Mann–Whitney U test, as applicable. Apgar scores were analyzed using the Friedman test for repeated measures. All statistical tests were two-sided, and statistical significance was set at *p* < 0.05. Analyses were conducted using Stata Statistical Software, version 16 (StataCorp LLC, College Station, TX, USA, 2019).

## 3. Results

**Figure 1:**
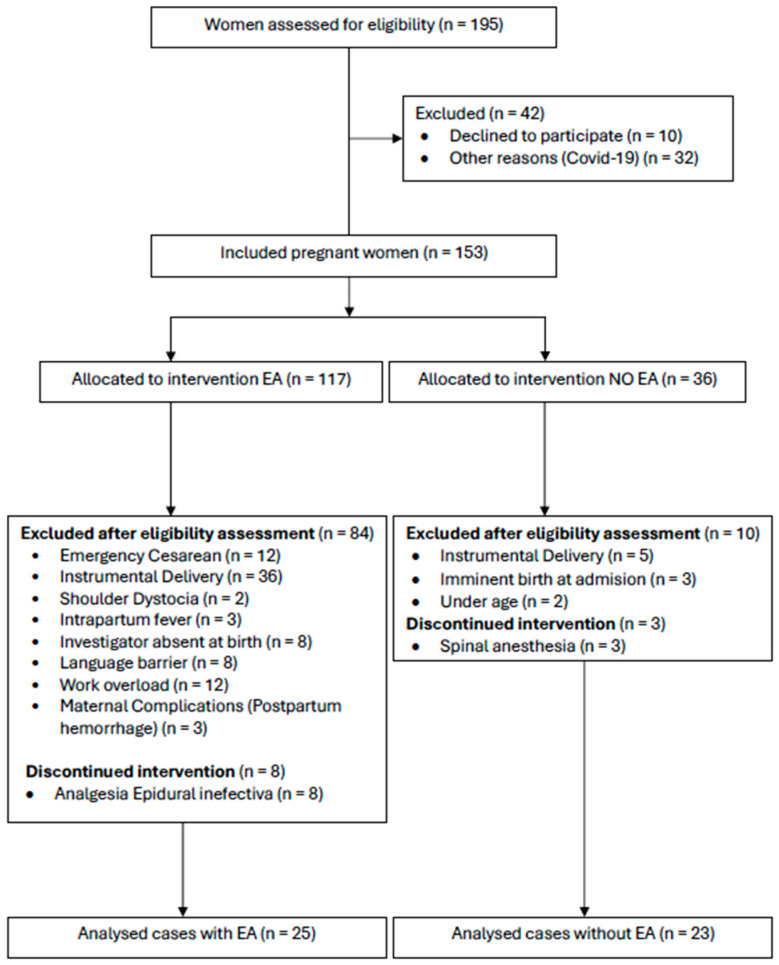
Flow diagram of study participants. The diagram shows the identification, eligibility assessment, inclusion and analysis of participants in the study. Reasons for exclusion at each stage are detailed according to STROBE recommendations for observational studies.

A prospective comparative observational cohort study included 48 term newborns at the Complejo Asistencial Universitario de Salamanca during the study period, from an initial sample of 195. A total of 147 newborns were excluded for not meeting the inclusion criteria. All newborns were selected from primiparous women at term in low-risk labor. Ultimately, 25 term newborns whose deliveries were performed with epidural analgesia (EA group) and 23 term newborns whose deliveries were performed without epidural analgesia (NE group) were assigned, as shown in the flow diagram of study participants (Figure 1).

Demographic data, as well as those related to the evolution of labor, are presented in Table 2 and Table 3.

No differences were observed between the epidural and non-epidural groups in NIRS parameters (CrSO_2_, cFTOE, cTOE), pCO_2_, pO_2_, SpO_2_, hemoglobin, blood glucose, or pH, and there were likewise no differences between groups in Apgar scores (Table 4 and Table 5).

15 min after birth, cerebral oximetry values in the epidural group (EA) were as follows: CrSO_2_ 79.5% ± 7.6, cFTOE 0.2 ± 0.1, and cTOE 17.6% ± 7.7; and in the non-epidural group (NE): CrSO_2_ 78.7% ± 10.3, cFTOE 0.2 ± 0.1, and cTOE 18.7% ± 10.8 (Table 5).

**Figure 2:**
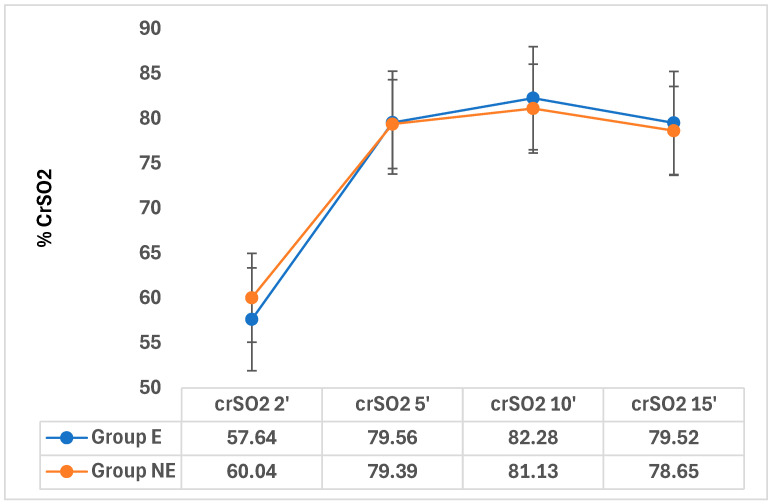
Cerebral oximetry values 15 min after birth.

Mean CrSO_2_ values [95% CI] increased over time in both groups. In the epidural group, CrSO_2_ rose from 57.64% at 2 min to 79.56% at 5 min and 82.28% at 10 min, remaining stable at 15 min (79.5%). A comparable trend was observed in the non-epidural group, increasing from 60.0% to 79.39% and 81.13% at 5 and 10 min, respectively, with stabilization at 15 min (78.65%). No statistically significant intergroup differences were detected at any time point (*p* > 0.05) (Table 5). CrSO_2_ exhibited the same pattern in both groups—namely, a gradual increase, a peak at 10 min, and stabilization thereafter at 15 min (Figure 2).

Statistically significant differences were identified in two obstetric parameters between groups: the duration of the passive phase of the second stage of labor (*p* = 0.001), which does not occur in deliveries without epidural analgesia, and the use of oxytocin during labor (*p* = 0.040), which was more frequent in epidural deliveries (52%) than in deliveries without epidural analgesia (21.7%) (Table 3). On the other hand, regardless of the total dose of local anaesthetic and opioid administered, no woman experienced hypotensive episodes or required vasoactive drugs, and motor block did not exceed Bromage grade I in any case.

No correlations were found in either group between NIRS parameters and any maternal variable, labor progression parameter, or analytical variables (hemoglobin, pO_2_, pCO_2_, blood glucose) that influence cerebral oxygenation. Correlation analyses are presented in Table 6.

## 4. Discussion

The aim of the present study was to evaluate the potential correlation between epidural analgesia and CrSO_2_, cFTOE, and cTOE (measured with NIRS), as well as with Apgar scores, umbilical cord blood gas parameters (pH, partial pressure of O_2_, partial pressure of CO_2_), and other metabolic variables in term neonates from low-risk deliveries at 15 min after birth. Research on the effect of epidural analgesia during labor on neonatal outcomes is important because the detailed influence of this association remains limited and debated, particularly as observational studies report mixed results, with some identifying associations with adverse neonatal outcomes and others finding no such link [6,7,8,9]. Epidural analgesia in labor, although highly effective and improving the maternal stress response, can affect the fetus through transplacental drug transfer and indirectly via changes in maternal and fetal physiology such as hypotension, reduced mobility, pruritus, maternal fever, fetal heart rate abnormalities, as well as an increased risk of assisted or operative vaginal delivery. All these conditions may contribute to adverse neonatal outcomes.

Most studies assessing the influence of epidural analgesia on immediate neonatal well-being evaluate Apgar scores, the need for resuscitation or transfer to the neonatal intensive care unit (NICU), or umbilical cord blood pH values. Overall, the majority of prospective, retrospective, and cohort studies report no clinically relevant differences in neonatal outcomes between deliveries with and without epidural analgesia, consistently demonstrating comparable Apgar scores and low rates of neonatal asphyxia [19,20,21].

Systematic reviews and meta-analyses similarly show no clear association between epidural analgesia and adverse neonatal outcomes, although the quality of evidence has been limited by methodological heterogeneity, imprecision in effect estimates, and potential publication bias [22]. Variations in the timing of epidural initiation [23], maintenance during the second stage of labor [24], or mode of epidural administration have likewise not been associated with differences in neonatal condition at birth [25,26]. Furthermore, neonatal outcomes appear comparable when different local anesthetics are used in low-concentration epidural regimens [27].

Nevertheless, some observational studies have reported lower Apgar scores or higher NICU admission rates among neonates exposed to epidural analgesia [8,28,29,30]. These findings remain inconsistent and are likely influenced by confounding factors related to labor complexity rather than a direct pharmacological effect of epidural analgesia. Maternal fever, prolonged labor, abnormal fetal presentation, increased use of instrumental delivery [31], and higher rates of obstetric interventions such as oxytocin augmentation have been proposed as contributing factors [32].

Taken together, the current literature suggests that the relationship between neuraxial labor analgesia and immediate neonatal outcomes remains heterogeneous and potentially confounded by clinical and organizational variables. Careful consideration of patient-, provider-, and hospital-level factors is therefore essential when interpreting observational data on neonatal outcomes associated with epidural analgesia [17].

Taking the above into account, the present study evaluated the influence of epidural analgesia on neonatal outcomes in term newborns from low-risk deliveries. There were no differences in Apgar scores at 1, 5, and 10 min, nor in umbilical cord blood gas values (pH, pO_2_, pCO_2_, and base excess). In neither group was neonatal resuscitation required, nor was there any need for NICU transfer.

The use of NIRS to assess neonatal cerebral oxygenation in the immediate postnatal period has been widely adopted because it enables continuous monitoring, allowing early detection of hypoxia and guiding timely therapy to reduce the risk of brain injury. Even today, there remains a knowledge gap regarding cerebral oxygenation patterns during the immediate neonatal transition.

Multiple NIRS studies have evaluated reference ranges for cerebral oxygen saturation during neonatal transition in term and preterm newborns, considering the technology used for measurement. Most studies, as in the present work, used INVOS. CrSO_2_ is on average 22.5% lower than peripheral saturation in preterm newborns and 20.6% lower in term newborns. In preterm newborns, CrSO_2_ stabilizes at 70–77% between 10 and 15 min after birth, whereas in term newborns it reaches 72–85%, results similar to those observed in both groups of our study [33].

Likewise, many studies have explored the influence of physiological parameters on cerebral oxygenation during neonatal transition. Hemodynamic adaptations appear to play a central role, with cerebral oxygenation evolving in parallel with cardiovascular changes occurring after birth. Noori et al. [34] have described associations between cerebral oxygenation and parameters such as cerebral blood flow, heart rate and ductal shunting, although other authors [35] have not found a statistically significant correlation between cardiac output and the cerebral tissue oxygenation index (cTOI).

Wolsfberger et al. [18] analyzed the influence of pCO_2_ on cerebral oxygenation, finding different correlations in term versus preterm newborns, in whom higher pCO_2_ values correlated negatively with CrSO_2_. In our study, there was no statistically significant correlation between pCO_2_ values and cerebral oximetry indicators in either the epidural analgesia group or the control group. Mattersberger et al. [36] analyzed relationships between metabolic parameters and CrSO_2_ and cFTOE. pH, base excess, and lactate are lower in preterm newborns, with a negative correlation of lactate with CrSO_2_ and a positive correlation with cFTOE, while only a positive correlation between cFTOE and bicarbonate was found in term newborns. The same author [37] observed a significant negative correlation between blood glucose and CrSO_2_, more pronounced in preterm than in term newborns, explained by vasodilation secondary to low glucose levels.

In the present study, no metabolic or hematimetric parameter correlated with cerebral oxygenation indicators in either group of newborns.

Several studies have specifically examined the influence of delivery mode on neonatal cerebral oxygenation during the immediate transition period [38,39,40,41,42]. Healthy newborns delivered by cesarean section comprised the most studied group. Although results are not entirely uniform, evidence suggests that the mode of delivery may produce transient differences in cerebral oxygenation patterns during the first minutes after birth. Vaginal delivery has been associated with higher cerebral blood volume and progressive increases in cerebral oxygen saturation [41,42], likely reflecting physiological cardiovascular and respiratory adaptation promoted by labor-induced stress, catecholamine release, and gradual lung fluid clearance.

In contrast, neonates born by cesarean section, particularly elective procedures without labor, may exhibit different early adaptation dynamics. Some studies [43] report higher cerebral oxygen saturation values and lower cerebral fractional tissue oxygen extraction shortly after birth, possibly related to reduced metabolic demand or altered pulmonary transition. Other investigations have described an initial decline or slower increase in cerebral oxygenation compared with vaginal births. Despite these early variations, most studies demonstrate convergence of cerebral oxygenation values within the first minutes of life, suggesting effective compensatory mechanisms that preserve cerebral oxygen balance regardless of delivery mode.

Assisted vaginal deliveries, including vacuum extraction, have also been evaluated, showing transient differences in cerebral oxygenation indices and tissue hemoglobin content immediately after birth, but without persistent clinically relevant effects [40]. Importantly, several investigations [38,39] have reported no significant differences in cerebral oxygenation parameters across delivery modes when assessed beyond the earliest transitional phase.

Overall, current evidence indicates that while delivery mode may influence the trajectory of early cerebral adaptation, it does not appear to result in sustained impairment of neonatal cerebral oxygenation in healthy term newborns.

Regarding the effect of maternal anesthesia on cerebral oxygenation measured by NIRS, studies are scarce and mainly focus on cesarean sections comparing general and spinal anesthesia [11,12,13,14,15,16]. In general, the differences observed seem to be related more to maternal hypotension and its management than to the anesthetic technique per se.

Despite extensive research evaluating the effects of epidural analgesia on conventional neonatal outcomes, current evidence remains limited to indirect clinical indicators of neonatal well-being. No previous studies have explored the potential influence of epidural analgesia on neonatal cerebral oxygenation, an objective physiological parameter reflecting early cerebral adaptation after birth. In this context, the present study provides novel data by assessing neonatal CrSO_2_ following delivery in relation to maternal epidural analgesia exposure. Our findings demonstrate comparable cerebral oxygenation patterns between neonates born to mothers with and without epidural analgesia, supporting the notion that epidural analgesia does not adversely affect early neonatal cerebral oxygenation. These results contribute new physiological evidence to the ongoing debate regarding the neonatal safety of epidural analgesia and may help refine the assessment of neonatal well-being beyond traditional clinical outcomes.

### Limitations

The present study has limitations and strengths. The main limitation is the sample size, particularly in the control group without epidural analgesia, due to the difficulty of accessing this population, as women who decline epidural analgesia during labor are a minority in our healthcare setting.

This study provides methodological homogeneity, as it was designed as a prospective cross-sectional study with rigorous population selection (term neonates born after vaginal delivery), thereby reducing population variability and avoiding centre-related differences in clinical practice. In addition, outcome measures with high interobserver variability, such as the Apgar score, were assessed by the same investigator using a standardized approach.

However, the strict selection of a homogeneous low-risk population limits the generalisability of the findings, and the results cannot be extrapolated to higher-risk populations.

The absence of serial monitoring of key variables, such as peripheral oxygen saturation (SpO_2_), haemoglobin levels, blood glucose, or blood gas analysis, represents an important limitation, as it prevented assessment of potential temporal associations between these parameters and changes in cerebral regional oxygen saturation (CrSO_2_).

Cerebral blood flow and cerebral blood volume were not assessed, which may have limited a more comprehensive evaluation of cerebral autoregulation. Nevertheless, previous NIRS studies have reported no significant differences in cerebral regional oxygen saturation during the first minutes after birth between cesarean and vaginal delivery in stable term neonates, suggesting that cerebral blood flow autoregulation may be largely independent of the mode of delivery.

There are no studies of this kind that evaluate, using NIRS, neonatal cerebral oximetry to assess the effect of epidural analgesia during vaginal deliveries; we expect this strength to be relevant for clinical practice and therapeutic decision-making during the neonatal transition.

## 5. Conclusions

In low-risk deliveries with term newborns, epidural analgesia does not alter neonatal cerebral oximetry (CrSO_2_) measured by NIRS, and the evolution of cerebral oximetry as shown by NIRS follows a pattern parallel to that of other fetal well-being parameters.

## Figures and Tables

**Table 1:** Epidural analgesia treatment regimens: infusion parameters according to drug concentration and analgesic technique.

Drug and Concentration	Technique	Continuous Rate	PCEA ^1^ Dose	Lockout Interval	PIEB ^2^ Regimen
Levobupivacaine 0.0625% + fentanyl 2 µg/mL	Continuous + PCEA	8 mL/h (2–12 mL/h)	6 mL (2–8 mL)	20 min	—
Levobupivacaine 0.0625% + fentanyl 2 µg/mL	Continuous + PCEA + PIEB	1 mL/h (0–2 mL/h)	6 mL (2–8 mL)	20 min	6 mL every 30 min (4–10 mL)
Levobupivacaine 0.125% + fentanyl 2 µg/mL	Continuous + PCEA	6 mL/h (2–10 mL/h)	5 mL (2–6 mL)	20 min	—
Levobupivacaine 0.125% + fentanyl 2 µg/mL	Continuous + PCEA + PIEB	1 mL/h (0–2 mL/h)	4 mL (2–6 mL)	20 min	5 mL every 30 min (2–8 mL)

^1^ PCEA: patient-controlled epidural analgesia; ^2^ PIEB: programmed intermittent bolus. Initial bolus consisted of 10 mL of levobupivacaine 0.125% plus fentanyl 5 µg/mL administered in fractions.

**Table 2:** Demographic and anthropometric data.

	Epidural Group *n* = 25	Non-Epidural Group *n*= 23	*p* Value ^4^
Maternal age (years)	31 (29–36) ^1^	31 (25–35)	0.71
Maternal weight (kg)	72.0 (61.0–84.0)	68.0 (65.9–79.0)	0.58
Maternal size (m)	1.62 (1.59–1.65)	1.64 (1.61–1.67)	0.31
Maternal BMI ^2^ (kg/m^2^)	28.0 (24.4–31.2)	25.6 (23.88–28.7)	0.32
Parity (*n*)	1 (1–1)	1 (1–1)	0.94
Gestational age (weeks)	39.74 (39.36–40.10)	39.54 (39.10–39.98)	0.49
NB ^3^ gender			0.78
- Female, *n* (%)	12 (48.0%)	10 (43.5%)	
- Male, *n* (%)	13 (52%)	12 (56.5%)	
NB weight (g)	3317.6 (3163.62–3471.58)	3121.96 (2984.11–3259.8)	0.058

^1^ Data are presented as mean (95%CI), median (IQR), or *n* (%). ^2^ BMI, body mass index; ^3^ NB, newborn. ^4^ A *p* value indicates a significant difference between the parameters of the NB group with and without epidural analgesia.

**Table 3:** Obstetric data.

		Epidural Group *n* = 25	Non-Epidural Group *n* = 23	*p* Value
Active phase of the 1st stage of labor, min		300.0 (210.0–360.0)	195.0 (165.0–360.0)	0.15
Latent phase of the 2nd stage of labor, min		30.0 (0.0–120.0)	0.0 (0.0–0.0)	<0.001 *
Active phase of the 2nd stage of labor, min		41.0 (20.0–60.0)	36.0 (18.0–60.0)	0.50
Amniorrhexis (time)				1.0
	<12 h	21 (84.0%)	20 (87.0%)	
	>12 h	4 (16.0%)	3 (17.0%)	
Amniorrehexis				0.22
	Spontaneous	19 (76.0%)	13 (56.5%)	
	Artificial	6 (24.0%)	10 (43.5%)	
Amniotic Fluid Color				1.0
	Clear	22 (88.0%)	20 (87.0%)	
	Meconium	3 (12.0%)	3 (13.0%)	
Group B Streptococcus				0.33
	Unknown	1 (4.0%)	0 (0.0%)	
	Positive	5 (20.0%)	2 (8.7%)	
	Negative	19 (76.0%)	21 (93.3%)	
Antibiotic Prophylaxis				0.73
	Yes	6 (24.0%)	4 (17.4%)	
	No	19 (76.0%)	19 (82.6%)	
Oxytocin				0.04
	Yes	13 (52.0%)	5 (21.7%)	
	No	12 (48.0%)	18 (78.3%)	

Data are presented as mean (95% CI), median (IQR), or *n* (%). * *p* value indicates a significant difference between the parameters of the newborn group with epidural analgesia and without epidural analgesia.

**Table 4:** Immediate neonatal assessment data.

	Epidural Group *n* = 25	Non-Epidural Group *n* = 23	*p* Value
pH	7.26 (7.23–7.30)	7.27 (7.23–7.31)	0.74
pO_2_, mm Hg	17.67 (15.66–19.67)	16.35 (14.30–18.39)	0.34
pCO_2_, mm Hg	51.40 (47.12–55.68)	50.18 (45.41–54.95)	0.69
Base excess, mEq/L	−4.84 (−5.90–−3.78)	−5.01 (−6.76–−3.25)	0.86
SpO_2_ (%)	97.20 (96.65–97.75)	97.30 (96.67–97.94)	0.80
Hb (g/dL)	16.67 (16.21–17.13)	16.49 (15.72–17.27)	0.67
Glycemia (mg/dL)	80.0 (70.0–92.0)	83.5 (68.5–92.0)	0.99
APGAR at 1 min	9 (9–9)	9 (9–9)	0.95
APGAR at 5 min	10 (10–10)	10 (10–10)	0.17
APGAR at 10 min	10.0 (10.0,10.0)	10 (10–10)	-

Data are presented as mean (95%CI), median (IQR), or No. (%). Hb, hemoglobin; pCO_2_, partial pressure of carbon dioxide; pO_2_, partial pressure of oxygen; SpO_2_, peripheral arterial oxygen saturation. The *p* value indicates a significant difference between the parameters of the newborn group with and without epidural analgesia.

**Table 5:** NIRS measurements.

Cerebral Regional Oxygen Saturation	Epidural Group *n* = 25	Non-Epidural Group *n* = 23	*p* Value
crSO_2_ 2 min, %	57.64 (51.52–63.76)	60.04 (53.80–66.29)	0.57
crSO_2_ 5 min, %	79.56 (75.06–84.06)	79.39 (74.10–84.68)	0.96
crSO_2_ 10 min, %	82.28 (78.93–85.63)	81.13 (76.93–85.33)	0.66
crSO_2_ 15 min, %	79.52 (76.40–82.64)	78.65 (74.21–83.09)	0.74
cFTOE 15 min, %	0.18 (0.15–0.21)	0.19 (0.14–0.24)	0.72
cTOE 15 min, %	17.64 (14.48–20.80)	18.65 (13.98–23.32)	0.71

Data are presented as mean (95%CI), median (IQR), or *n* (%). NIRS, near-infrared spectroscopy; crSO_2_, cerebral regional oxygen saturation; cFTOE, cerebral fractional tissue oxygen extraction; cTOE, cerebral tissue oxygen extraction; *p* value indicates significant difference between the parameters of the newborn group with epidural analgesia and without epidural analgesia.

**Table 6:** Correlations.

	crSO_2_ ^1^ 15 min			crSO_2_ 15 min		
	Epidural Group			Non-Epidural Group		
	*n*	r	*p*-Value	*n*	r	*p*-Value
Maternal age	25	0.0782201	0.801	23	−0.0643454	0.856
Gestational age	25	−0.2099331	0.906	23	1.808551	0.414
NB weight	25	−0.0019444	0.648	23	−0.0096081	0.167
SpO_2_ 15	25	−0.1809524	0.881	23	−2.124304	0.161
Hb	25	1.810193	0.253	23	3.830929	0.006
Glycemia	25	−0.1181868	0.097	23	−0.1627571	0.158
pH	25	−16.0952	0.401	23	−15.47619	0.537
pO_2_	25	−0.093482	0.794	23	0.0019308	0.997
pCO_2_	25	0.0920991	0.547	23	0.0908241	0.675
Base excess	25	−0.5419074	0.453	23	−0.2027555	0.787
Apgar 5 min	25	5.456522	0.338	-	-	-
Oxytocin	25	6.852564	0.02	23	2.366667	0.659

^1^ crSO_2_: Cerebral regional oxygen saturation.

## Data Availability

Detailed data are available upon reasonable request to the corresponding author.

## Appendix A

**Table A1:** Exclusion criteria.

Maternal Medical History	Risk Factors in Current Pregnancy	Intrapartum Complications
- Hypertension - Heart disease - Renal disease - Diabetes mellitus and other endocrinopathies - Chronic respiratory disease - Hematologic disease - Epilepsy and other neurologic disorders - Psychiatric disease - Liver disease - Autoimmune disease - Severe medical–surgical pathology	- Hypertensive disorders of pregnancy - Severe anemia - Gestational diabetes - Perinatally transmissible infection - Rh isoimmunization - Multiple pregnancy - Polyhydramnios or oligohydramnios - Vaginal bleeding - Placenta previa - Abnormal fetal growth - Congenital fetal anomaly - Preterm prelabor rupture of membranes	- Obstetric emergency (hemorrhage, placental abruption, or cord prolapse) - Inadequate labor progression - Induction of labor - Instrumental delivery - Cesarean delivery - Pathological cardiotocography (CTG) - Maternal fever > 38 °C - Rupture of membranes > 24 h, Birth weight < 2500 g - Need for respiratory support or resuscitation - Admission or transfer to the neonatal intensive care unit
